# Pleural MAC30 as a prognostic marker in NSCLC with malignant pleural effusion

**DOI:** 10.18632/oncotarget.22631

**Published:** 2017-11-22

**Authors:** Yi Shan, Hui Ding, Junjie Lu, Zhijun Ge, Yongfei Tan

**Affiliations:** ^1^ Department of Critical Care Medicine, The Affiliated Yixing Hospital of Jiangsu University Yixing, Jiangsu 214200, China; ^2^ Department of Respiratory, The Affiliated Yixing Hospital of Jiangsu University Yixing, Jiangsu 214200, China; ^3^ Department of Cardiac & Thoracic Surgery, The Affiliated Yixing Hospital of Jiangsu University Yixing, Jiangsu 214200, China

**Keywords:** MAC30, MPE, NSCLC, BPE, OS

## Abstract

Over-expressed meningioma-associate protein (MAC30) in tissues was associated with malignant tumor differentiation, metastasis and poor prognosis. However, the attention of MAC30 in pleural effusion from lung tumor is insufficient. Our retrospective study was prepared to explore the clinical values on diagnosis and prognosis of MAC30 from malignant pleural effusion (MPE) in non-small cell lung cancer (NSCLC). Levels of MAC30 were confirmed in MPE from 48 NSCLC patients and in benign pleural effusion (BPE) from 45 controls via enzyme-linked immunosorbent assay (ELISA). The association of MAC30 in MPE with clinical significance was further determined. We found that the levels of MAC30 in MPE were obviously higher than those in BPE (p < 0.05). Moreover, with a cutoff point (17.5 ng/ml), we confirmed the sensitivity and specificity of MAC30 for MPE were 82.7% and 85.3% using ROC curve analysis. Indeed, longer overall survival (OS) was present in NSCLC patients with low MAC30 expression in MPE. Multivariate analysis explicated that elevated MAC30 in MPE was an independent prognostic factor for shorter OS of NSCLC. Our data suggests that MAC30 in pleural effusion could be a potential prognostic marker in NSCLC with MPE.

## INTRODUCTION

Lung malignancies stably control the leading cause of cancer-related death worldwide even with diagnostic and clinical therapeutic advances [1]. Accounting for more than 80% of lung cancers, non-small cell lung cancer (NSCLC) always shows metastases to distant sites at the time of diagnosis [2]. As a common clinical complication in more than 20% of NSCLC, Malignant Pleural Effusion (MPE) as an independent factor deteriorates the condition of patients and induces the poor survival [3]. Once the definition of MPE in NSCLC is confirmed, patients will be classified into advanced stages in TNM with a median survival of 5.5 months [4]. Thus, the distinction between MPE and benign pleural effusion (BPE) from cytological or histological diagnosis via thoracocentesis, pleural biopsy, and thoracoscopy is the first and critical step in clinical intervention in order to plan early initiation and suitable management. But, unfortunately, part of NSCLC patients with MPE always experience unclear diagnosis with cytological negative. Indeed, the detection of several tumor biomarkers such as carcinoembryonic antigen (CEA), cytokeratin 19 fragments (CYFRA 21-1) and carbohydrate antigen 153 (CA153) in pleural effusion is assisted to get proper diagnosis for patients with a deep suspicion of malignancy [5–7]. However, because of the unsatisfied diagnostic accuracy from 30 to 60%, these biomarkers limit their clinical application value [8, 9]. Therefore, it is essential to select an accurate biomarker for early detection of MPE and predicting prognosis of lung malignances.

Previous reports indicated that Meningioma-associated protein (MAC30) locating on 17q11.2 [10] showed variously expression in different malignances, as over-expressed in breast, esophagus, lung and colon cancers [11–13] while down-expressed in pancreatic and renal cancers [14]. In deed, in our previous study, we already confirmed the elevated level of MAC30 in lung tissues from patient with NSCLC as a useful biomarker for poor prognosis and resistance to platinum-based chemotherapy [13, 15]. However, it's unclear about the relationship of MAC in MPE with the clinical investigation in diagnosis and prognosis of NSCLC patients.

In this present study, we hypothesized that MA30 level was increased in NSCLC-induced pleural effusion. Moreover, we explored the clinical value of MAC30 in MPE on diagnostic and prognostic instructions.

## RESULTS

### Pleural MAC30 expression in NSCLC

**Figure 1:**
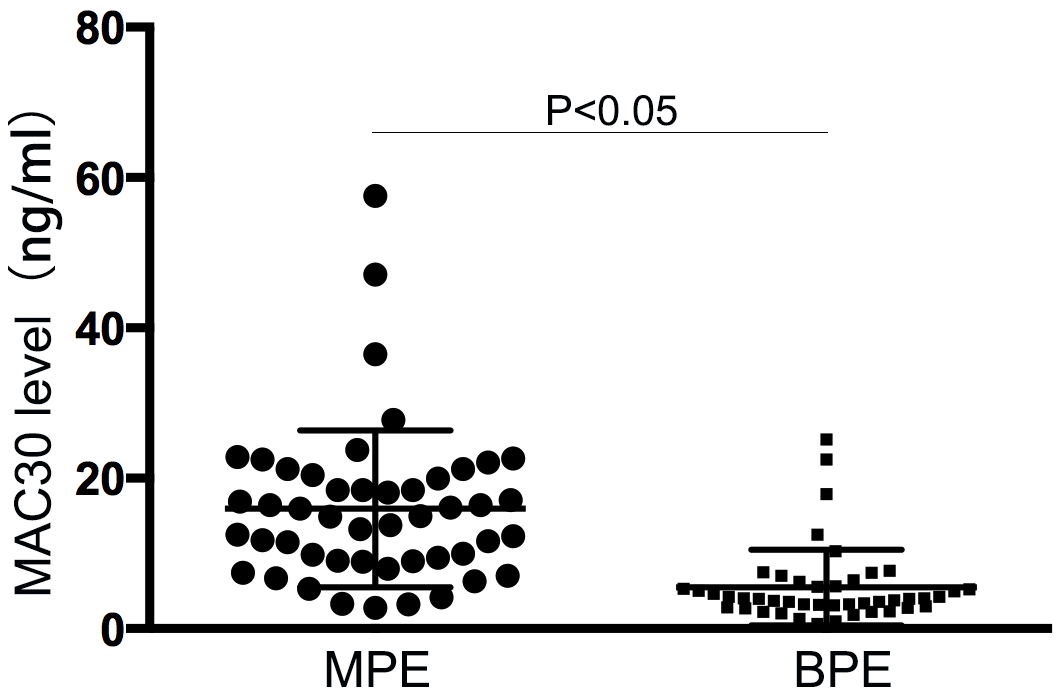
The levels of MAC30 in pleural effusion Elevated MAC30 levels in MPE was presented than those in BPE (*p* < 0.05).

The characteristics of a total of 48 NSCLC with MPE and 45 controls in the present study were summarized in Table 1. There's no statistical difference in age, gender and smoking history between the two groups. As shown in Figure 1, MAC30 level in MPE from NSCLC detected via ELISA was significantly increased than that in BPE (18.25 ± 6.11 ng/ml vs. 5.07 ± 0.72ng/ml, *p* <0.05) (Figure 1).

**Table 1:** Various clinicopathological features of patients

Variables	MPE	BPE	*p* value
No.	48	45	
Age (years)			0.773
<59	31	26	
≥59	17	19	
Gender			0.586
Male	26	21	
Female	22	24	
Smoking status			0.612
Non-smoker	31	28	
Smoker	17	17	
Histological type			
SQCLC	11	ND	
AC	37	ND	
AS	0	ND	
BPE			
Tuberculous	ND	9	
Parapneumonic	ND	28	
Others	ND	8	

MPE, malignant pleural effusion; SQCLC, squamous cell carcinomas; AC, adenocarcinoma; AS, adenosquamous; BPE, benign pleural effusion; ND, no data;

### Clinical diagnostic role of MAC30 on MPE

**Figure 2:**
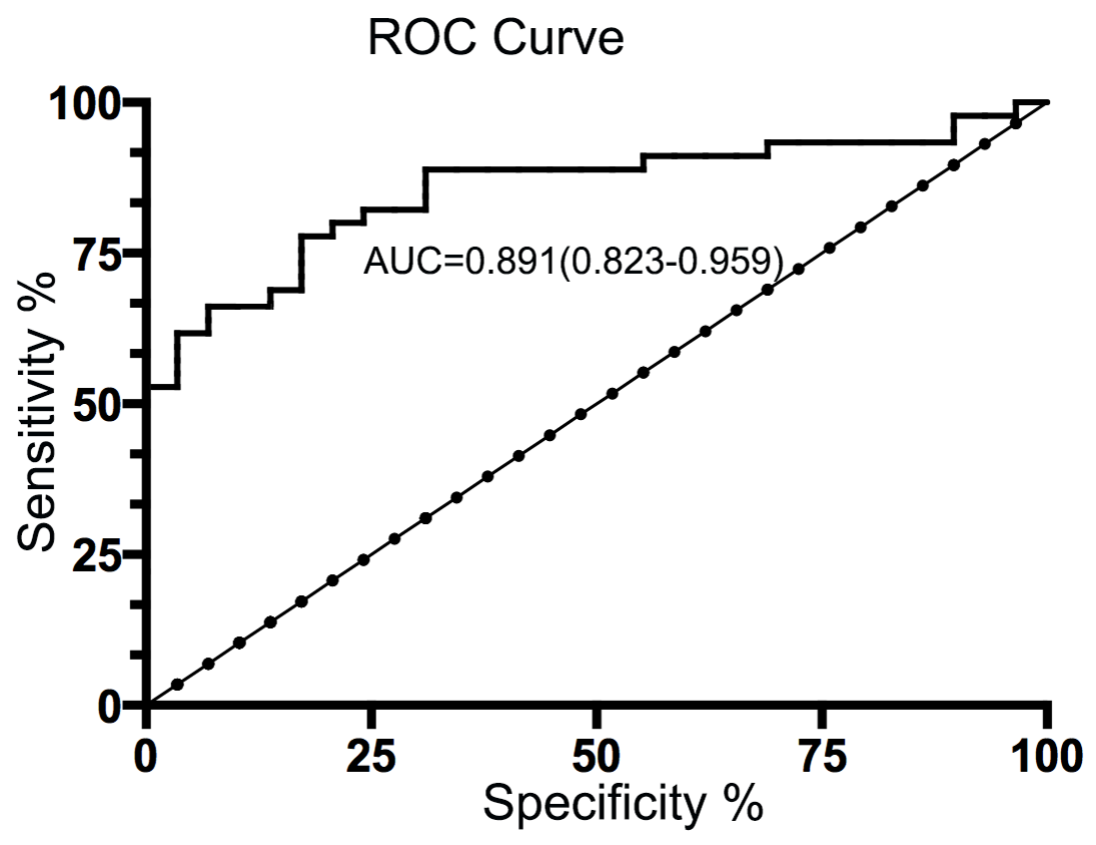
Diagnostic value of pleural fluid MAC30 levels for NSCLC patients with MPE Based on a cutoff value of 17.5 ng/ml according to pleural fluid MAC30 concentrations (AUC=0.891; 95% CI=0.823–0.959), the ROC curve discrimination of MPE and BPE presents the sensitivity 82.7% and specificity 85.3%.

In order to identify the clinical value of MAC in MPE, we prepared ROC curve with a cutoff point of MAC30 level (17.5 ng/ml) to ensure the diagnostic threshold that the area under the curve (AUC) was 0.891 (95% CI 0.823–0.959) in Figure 2. Based on the judgment, MAC30 made a considerable sensitivity 82.7% and specificity 85.3%.

### Pleural MAC30 level and clinicopathological elements in NSCLC with MPE

The relationship between pleural MAC30 level and characteristics of NSCLC patients with MPE was shown in Table 2. As expected, MAC30 concentration in MPE was associated with distant metastasis, but not with age, gender, histological type and smoking. Furthermore, higher level of MAC30 in MPE was found in advanced NSCLC patients with metastasis to brain or bone.

**Table 2:** MAC30 in MPE with the clinicopathological factors of NSCLC patients

Variables	Number	MAC30 (ng/ml)	*p* value
Age (years)			0.518
<59	31	13.556	
≥59	17	14.731	
Gender			0.275
Male	26	15.648	
Female	22	16.994	
Smoking status			0.887
Non-smoker	31	15.937	
Smoker	17	13.728	
Histological type			0.665
SQCLC	11	18.664	
AC	37	15.832	
Distant metastasis			**0.013**
Absent	22	25.741	
Present	26	12.446	

SQCLC, squamous cell carcinomas; AC, adenocarcinoma;

### Prognostic value of MAC30 in MPE for advanced NSCLC patients

**Figure 3:**
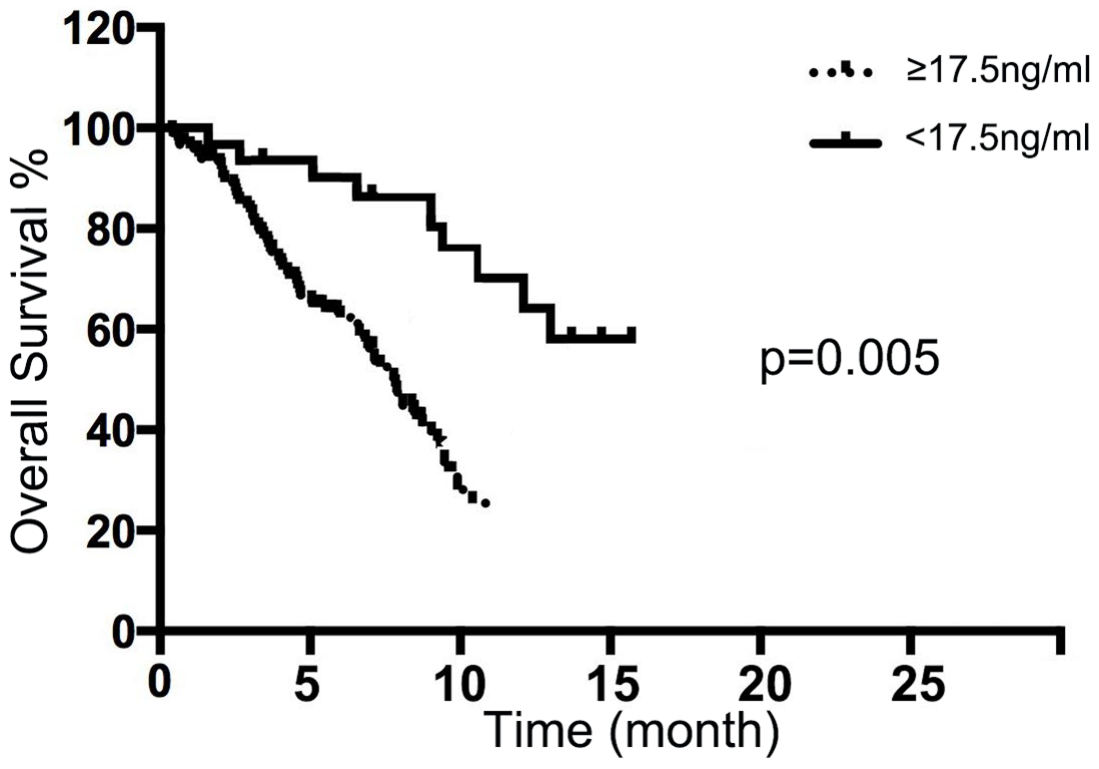
Pleural fluid MAC30 expression with overall survival of NSCLC patients The NSCLC patients with high MAC30 expression had a significantly worse outcome compared with the patients with low MAC30 level (*p* = 0.005).

According to the cutoff point (17.5 ng/ml), advance NSCLC patients with high expression of MAC30 in MPE were suffered with poorer OS via Kaplan-Meier curve analysis (p<0.05) (Figure 3). Furthermore, the univariate and multivariate analyses indicated that MAC30 expression was an independent prognostic biomarker for OS in patients with NSCLC (Table 3). So, MAC30 expression in pleural effusion could act as a novel tumor biomarker for the prognosis of advanced NSCLC.

**Table 3:** Univariate and multivariate analysis of prognostic factors in NSLCL patients

Variables	Univariate		Multivariate	
	HR	*P value*	HR	*P value*
MAC30 expression	1.373	**0.012**	1.627	**0.008**
Age (years)	1.736	0.551		
Gender	1.198	0.484		
Histological type	1.331	0.582		
Smoking status	1.236	0.433		
Distant metastasis	1.177	**0.007**	1.482	**0.005**

## DISCUSSION

In the present study, we firstly identified the elevated levels of MAC30 in pleural effusion from advanced NSCLC compared with those in BPE controls. Further data confirmed that pleural MAC30 could discriminate lung cancer from BPE with considerable sensitivity and specificity for diagnostic rationality of MPE. Moreover, we not only confirmed the positive association between pleural effusion MAC30 and distant metastasis in NSCLC, but also found the poorer OS in patients with higher levels of pleural MAC30. To the best of our knowledge, we firstly demonstrated that pleural effusion MAC30 was an independent prognostic marker for OS of advanced NSCLC. So, our findings imply that MAC30 in pleural effusion could act as a potential diagnostic marker for lung cancer-associated malignant effusion.

The diagnostic use of biomarkers for MPE is based on discriminating lung cancer from BPE with considerable sensitivity and specificity. Previous studies have already investigated the clinical value of several biomarkers in pleural fluid including (CEA), CYFRA21-1, CA125 and Romo1 [16–18]. Among those, CEA, CYFRA21-1 and CA125 were widely accepted in clinical performance as to distinguishing MPE from BPE. However, the real availability in clinical diagnostic of malignancies is doubtful. As a glycoprotein component of glycocalyx in the endothermic epithelium, CEA was confirmed to act as an biomarker for the diagnosis of MPE with a sensitivity from 29% to 82% and a specificity ranging from 77% to 93% [5]. Indeed, the increased expression of CEA in pleural fluid also could be found in non-malignant diseases, as inflammatory, lung tuberculosis and pulmonary fibrosis. CYFRA21-1 expressing especially in lung squamous cell carcinoma [17] yielded sensitivity from 22% to 91% and specificity from 80% to 97% [19]. Moreover, the previous study revealed that there was little clinical difference in identifying lung caner-related MPE between CEA and CYFRA21-1 [20]. A recent report suggested that concurrent use of pleural fluid CEA and Romo1 showed better diagnostic performance than did CEA or CYRFA 21-1 alone [17], but the sample in the study was relatively small. In this study, we found that higher level of MAC30 in pleural effusion presented an acceptable sensitivity and specificity for the diagnosis of MPE. In addition, pleural MAC30 associating with OS was an independent biomarker for prognosis of NSCLC with MPE. The clinical use of MAC30 as a promising non-invasive biomarker for NSCLC diagnosis and prognosis was viewed.

MAC30, as a member of the insulin-like growth factor-binding protein family (IGFBP), was approved to play as a suppressor or a promoter in different cancers. As a newly concerned gene, the biological influence of MAC30 in tumor metabolism remains unclear. Pervious study confirmed that enhanced MAC30 expression in oral squamous cell carcinoma was positive with lymph nodal metastasis and server clinical prognosis [21]. Indeed, our previous studies confirmed that NSCLC patients with higher MAC30 expression resisted to platinum-based chemotherapy and exhibited worse survival [13, 15]. It's difficult for NSCLC patients with pleural effusion to receive invasive procedures including needle pleural biopsy or thoracostomy in order to get cytological examination. Meanwhile, it's critical to clear that the pleural effusion in NSCLC is MPE or BPE, because MPE not only indicates advanced disease but also comes with poor prognosis. In clinical process, a lung cancer patient with pleural effusion is firstly classified into advance cancer stage. However, *Porcel* et al [22] demonstrated that pleural effusions in NSCLC also could be benignant especially combining with obstructive pneumonia or heart failure. Biomarker in pleural effusion was investigated to identify its nature associating with the progress of disease. However, the clinical use is limited in most markers [3]. Based on our previous findings in lung tissue, we hypothesized that the expression of MAC30 in MPE was elevated compared with that in BPE. To the best of our knowledge, our data firstly indicated that pleural fluid MAC30 was contributed to determine whether the effusion is malignant or benignant. Thus, it is clinically meaningful to select sensitive markers for malignant effusion.

The present study verified that MAC30 in pleural effusions presented as a clinical prognostic marker of OS in advanced NSCLC. However, the study also contains several limitations. First, the collected size was relatively small because of a retrospective study. The further step is to enroll more samples to confirm our points. Second, the paired serum samples were lacking. The both measurement of serum and pleural fluid MAC30 could provide us more information about the sensitive and reliable expression of MAC30 in determining pleural fluid etiology. Third, although we confirmed that MAC30 expression in lung tissue was related to both poor response and prognosis in NSCLC with platinum-based chemotherapy, the possible relationship between pleural fluid MAC30 with therapeutic response was not confirmed. Thus, further research is focused on the conclusion whether MAC30 in MPE could be a predictive factor for platinum-based therapy response.

In conclusion, our present data suggested that pleural MAC30 concentration showed acceptable diagnostic sensitivity and accuracy to predict whether a pleural effusion is benign or malignant. Moreover, the levels of MAC30 in MPE could reflect the prognosis of NSCLC. In addition, our study provided clues for future observation about the clinical implication of MAC30 in NSCLC with MPE.

## MATERIALS AND METHODS

### Patients and tissue samples

In our study, we prospectively enrolled 48 NSCLC patients with MPE who were admitted to the department of Respiratory and Critical Care Medicine at Yixing people hospital affiliated Jiangsu University from May 2011 and March 2016. And 45 BPE from non-cancer patients were collected as controls in our study. Patients who had received preoperative chemotherapy were excluded. The present study was approved by the ethics committees of Jiangsu University Faculty of Medicine. The informed consent obtained from all subjects was confirmed.

### Study subjects and specimens

Pleural effusion was collected from subjects and centrifuged at 3000rpm for 10min at 4°C, and then supernatant was frozen and stored at -80°C until analysis. According to the manufacturer's instructions of ELISA kit (Mlbio, Shanhai, China), we determined MAC30 expression in pleural effusion. All samples were checked in duplicates.

### Statistical analysis

All data was analyzed by SPSS version 17.0. The level of statistical significance was considered as p<0.05 for all tests. The diagnostic value of MAC3O was assessed with ROC curve. Moreover, overall survival (OS) was performed by Kaplan-Meier analysis following with the relationship with MAC30 levels. Several factors as age, gender, histological type, smoking index, histological type and MAC30 were assessed to confirm the significance for prognosis via univariable and multivariate Cox. Independent prognostic factors influencing OS were identified via a cox regression model.
